# What makes a host a good reservoir? Determinants of the reservoir potential of *Nicotiana glauca* for tobacco mild green mosaic virus

**DOI:** 10.1093/ve/veaf044

**Published:** 2025-06-06

**Authors:** Rafael de Andrés-Torán, Aurora Fraile, Sayanta Bera, Miguel Ángel Mora, Michael McLeish, Fernando García-Arenal

**Affiliations:** Centro de Biotecnología y Genómica de Plantas (CBGP UPM-CSIC/INIA), Universidad Politécnica de Madrid and E.T.S.I. Agronómica, Alimentaria y de Biosistemas, Campus de Montegancedo, UPM, Pozuelo de Alarcón, 28223, Madrid, Spain; Centro de Biotecnología y Genómica de Plantas (CBGP UPM-CSIC/INIA), Universidad Politécnica de Madrid and E.T.S.I. Agronómica, Alimentaria y de Biosistemas, Campus de Montegancedo, UPM, Pozuelo de Alarcón, 28223, Madrid, Spain; Centro de Biotecnología y Genómica de Plantas (CBGP UPM-CSIC/INIA), Universidad Politécnica de Madrid and E.T.S.I. Agronómica, Alimentaria y de Biosistemas, Campus de Montegancedo, UPM, Pozuelo de Alarcón, 28223, Madrid, Spain; Centro de Biotecnología y Genómica de Plantas (CBGP UPM-CSIC/INIA), Universidad Politécnica de Madrid and E.T.S.I. Agronómica, Alimentaria y de Biosistemas, Campus de Montegancedo, UPM, Pozuelo de Alarcón, 28223, Madrid, Spain; Centro de Biotecnología y Genómica de Plantas (CBGP UPM-CSIC/INIA), Universidad Politécnica de Madrid and E.T.S.I. Agronómica, Alimentaria y de Biosistemas, Campus de Montegancedo, UPM, Pozuelo de Alarcón, 28223, Madrid, Spain; Centro de Biotecnología y Genómica de Plantas (CBGP UPM-CSIC/INIA), Universidad Politécnica de Madrid and E.T.S.I. Agronómica, Alimentaria y de Biosistemas, Campus de Montegancedo, UPM, Pozuelo de Alarcón, 28223, Madrid, Spain

**Keywords:** conditional mutualism, maintenance host, across-host trade-offs, *Tobamovirus*, *Nicotiana glauca*

## Abstract

Identifying traits that make a host a good reservoir for virus emergence is central to understanding virus ecology, host range evolution and mitigating virus epidemics, but is often hindered by a lack of knowledge on the infection dynamics of the virus in the reservoir population. Here we analyse traits that determine the reservoir potential of the wild plant *Nicotiana glauca* for tobacco mild green mosaic virus (TMGMV), an important pathogen of pepper (*Capsicum annuum*) crops, using epidemiological, experimental and population genetic approaches. We show that TMGMV is maintained at high prevalence in *N. glauca* populations that share the space with pepper crops in South eastern Spain. High prevalence may be explained by low virulence associated with TMGMV behaving as a conditional mutualist, which is in part explained by increased survival of infected plants under drought conditions. We also show maintenance in *N. glauca* populations of TMGMV genotypes that have a within-host fitness advantage in pepper and a disadvantage in *N. glauca*. This is explained by pleiotropic effects of host range mutations that result in higher vertical transmission through the seeds of *N. glauca* of isolates adapted to pepper. Last, high migration from *N. glauca* prevents fixation of pepper-adapted genotypes in pepper populations. Our results underscore the need to analyse the effects of infection on a range of host life-history traits, and effects of host range mutations on different components of virus fitness, to understand dynamics of infection and virus host range evolution.

## Introduction

Understanding the interplay of factors that cause pathogen emergence is a major focus of research in disease ecology, because of the potentially high impact in human and animal health, agriculture, and conservation, of emerging infectious diseases (Woolhouse et al. 2001, Haydon et al. 2002, Anderson et al. 2004, Jones 2009, Elena et al. 2014). Emergent pathogens are often multi-host pathogens (Woolhouse et al. 2001, Haydon et al. 2002, Anderson et al. 2004) that spill-over onto a new host population (target population) from one or more reservoirs, a reservoir being defined as one or more epidemiologically connected populations in which the pathogen can be permanently maintained and from which infection is transmitted to the defined target population (Haydon et al. 2002). Hosts of multi-host pathogens differ in susceptibility and competence and hence they also differ in reservoir potential, that is, the relative ability to sustain pathogen populations for transmission to new hosts (Cronin et al. 2010, Martin et al. 2019, Welsh et al. 2020). Thus, identifying the determinants of host reservoir potential is central to understanding infection dynamics and host range evolution and predicting emergence.

Determinants of reservoir potential have been proposed on the basis of theory and model analyses (Haydon et al. 2002, Cronin et al. 2014, Viana et al. 2014, Martin et al. 2019, Nuismer et al. 2022), with varying empirical or experimental support. For example, factors intrinsic to the host, such as phylogenesis or life-history traits, are considered to determine reservoir potential (Cronin et al. 2010, Ostfeld et al. 2014, Han et al. 2015, Holmes 2022, Nuismer et al. 2022). There is evidence that phylogenetic relatedness between reservoir and target hosts is positively associated with reservoir potential (Moury et al. 2017, Holmes 2022, French et al. 2023). Also, reservoir potential has been associated with a short life span (Malmstrom et al. 2005, Cronin et al. 2010, Johnson et al. 2012, Hily et al. 2014), which in plants often involves a quick-return physiological phenotype, that is, high rates of photosynthesis and efficient conversion of nutrients, photosynthates, and other resources into growth (Wright et al. 2004). Less evidence is available on factors related to the epidemiological connectivity between the reservoir and host populations, or to the capacity of the reservoir population to maintain permanently the pathogen, which requires that the population is larger than the critical community size (Haydon et al. 2002, Viana et al. 2014). Critical community size, that is, the minimum size of a closed population within which a pathogen can persist indefinitely (Haydon et al. 2002) is not independent of pathogen virulence, defined as the negative effect of infection on host fitness (Alizon et al. 2009, Little et al. 2010), but low virulence in the reservoir has not been identified as a condition of reservoir potential (Haydon et al. 2002, Geoghegan and Holmes 2018, Holmes 2022). The identification of reservoirs is difficult (Viana et al. 2014), and reservoirs are often unknown for recently emerged viruses, such as SARS-CoV-2 (Markov et al. 2023). This is also the case for most plant viruses that emerged in the last 50 years (e.g. Fargette et al. 2006, García-Arenal and Zerbini 2019), although the role of wild plants in the epidemiology of viral diseases in crops has been a focus of attention for a long time (e.g. Carter 1961, Harrison 1981, Gibbs 1983, Cooper and Jones 2006, Jones 2018). One notable exception is the RNA virus tobacco mild green mosaic virus (TMGMV), a major pathogen of pepper (*Capsicum annuum* L. Solanaceae) crops that has a reservoir in the wild plant *Nicotiana glauca* Grah. (Solanaceae). Here we study the *N. glauca*-TMGMV system to test a set of hypotheses related to the factors that determine reservoir potential.

*N. glauca* is an evergreen bush that can live up to 10 years, and a native of the Eastern-Central Andes now widely distributed in semiarid regions of the world, including Spain, where it was introduced in the first half of the nineteen century (MITECO 2020, Vélez-Gavilán 2023). In the Mediterranean basin, *N. glauca* is visited by hymenopteran and lepidopteran insects that collect the nectar (MITECO 2020, Ollerton et al. 2012) and may contribute to the horizontal transmission of viruses (Darzi et al. 2018, Levitzky et al. 2019). In South eastern (SE) Spain, it has a patchy distribution in stands of 20–200 individuals, where a TMGMV prevalence of about 75% was reported in 1984–86 (Rodríguez-Cerezo et al. 1991). TMGMV belongs to the species *Tobamovirus mititessellati*, genus *Tobamovirus* (family *Virgaviridae*). Tobamoviruses are vertically transmitted through the seed, and horizontally by plant-to-plant contact (Dombrovsky and Smith 2017) and insect pollinators (Darzi et al. 2018, Levitzky et al. 2019). TMGMV was first isolated from *N. glauca* plants from the Canary Islands (McKinney 1929), and later was reported infecting plants of this species in California, India, the Middle East, Australia, and the Mediterranean basin (Randles et al. 1981, Fraile et al. 1996). Data on high prevalence, multiplication rates, and competitive ability with tobacco mosaic virus indicate that TMGMV is well adapted to *N. glauca* (Bald and Goodchild 1960, Randles et al. 1981, Rodríguez-Cerezo et al. 1991, Fraile et al. 1997). Since the 1980s, TMGMV has emerged as an important pathogen of pepper crops in the Mediterranean basin, causing a highly severe syndrome of systemic necrosis (Moury and Verdin 2012, Velasco et al. 2020). Phylogenetic analyses showed that TMGMV isolates from *N. glauca* and pepper cluster together (Bera et al. 2018). However, isolates from *N. glauca* multiply to higher levels in *N. glauca* than in pepper, while isolates from pepper multiply to similar levels in both hosts; that is, isolates from *N. glauca* are adapted to this host, while TMGMV isolates from pepper are not adapted to pepper (Bera et al. 2018), but the underlying mechanisms of this asymmetry have not been explored. A duplication in the 3′ untranslated region (3’UTR) of TMGMV genome is a host range determinant: in single infection, isolates with (Long-3’UTR) or without (Short-3’UTR) this duplication multiply to similar levels in *N. glauca*, but Short-3’UTR isolates multiply to higher levels in pepper than in *N. glauca* (Bera et al. 2018). However, in coinfection, Long-3’UTR isolates outcompete Short-3’UTR isolates in *N. glauca* (Bodaghi et al. 2004), indicating their higher fitness in this host. Both Long-3’UTR and Short 3’UTR isolates cocirculate in *N. glauca* populations of California and SE Spain, and in pepper crops in SE Spain (Bodaghi et al. 2004, Bodaghi et al. 2000, Bera et al. 2018). The available information suggests that the advantage conferred by the 3’UTR dimorphism in each host is countered by pleiotropic effects on other components of virus fitness, and/or that inoculum fluxes between the *N. glauca* and pepper populations are high enough to prevent the fixation of the most advantageous 3’UTR allele in each host population.

In this work, we analyse the factors that determine the potential of *N. glauca* as a reservoir of TMGMV. We test three hypotheses: (i) high prevalence of TMGMV in *N. glauca* populations is associated with low virulence, (ii) the 3’UTR polymorphism is maintained in *N. glauca* populations because of contrasting pleiotropic effects on different components of virus fitness, and (iii) TMGMV adaptation to pepper is prevented by migration between the *N. glauca* and pepper TMGMV populations. These hypotheses address the role of *N. glauca* as a maintenance host of TMGMV in general, and of isolates adapted to pepper in particular, and the connectivity between the *N. glauca* and pepper TMGMV populations, and thus relate to the role of *N. glauca* as a reservoir of TMGMV for emergence in pepper crops.

## Materials and methods

### Study area, plant sampling, and detection of *Tobamovirus* infection

**Figure 1:**
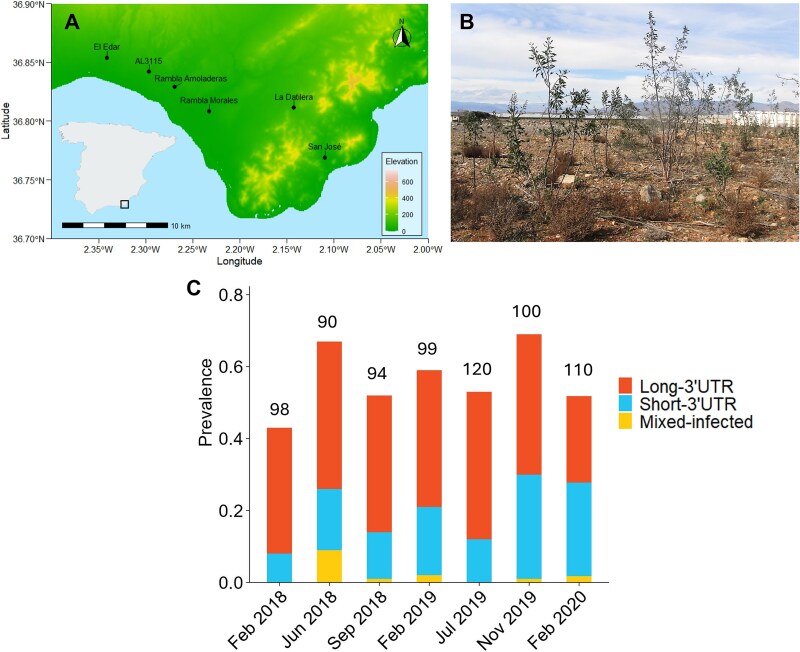
**A) Geographic location of the *N. glauca* populations in Almería monitored between February 2018 and 2020.** The six *N. glauca* populations located in SE Spain are shown, and the elevation is indicated at the bottom right. **B) Wild *N. glauca* plants from the La Datilera population. C) Variation of TMGMV prevalence in the six *N. glauca* populations.** The relative frequency of plants infected by Long-3’UTR and Short-3’UTR isolates, or mixed-infected by Long-3’UTR and Short-3’UTR isolates is shown for each collection time. The number of analysed plants is indicated above the columns.

Field work was performed between February 2018 and 2020 in the province of Almeria, SE Spain. Collections were conducted three times a year (5 February 2018, 19 June 2018, 14 September 2018, 18 February 2019, 11 July 2019, 8 November 2019, and 18 February 2020) at six *N. glauca* populations on a 60 km east–west transect. Population names and coordinates are: San José (36°46′07.4″N 2°06′34.5″W), La Datilera (36°48′40.8″N 2°08′32.2″W), Rambla Morales (36°48′29.7″N 2°13′57.3″W), Rambla Amoladeras (36°49′44.4″N 2°16′07.6″W), AL3115 (36°50′31.2″N 2°17′47.8″W), and El Edar (36°51′13.5″N 2°20′28.3″W) (Fig. 1  A, B). Plants were sampled at fixed distances on predetermined itineraries covering the extent of each population, regardless of plants showing symptoms of virus infection or not. All plants encountered along the itineraries were sampled in the smaller populations, and one out of three plants in the larger ones. The population extent and census varied from ~320 m^2^ and 20 plants (El Edar) to ~2000 m^2^ and 100–350 plants (San José, La Datilera). Plant positions were mapped, and older, noticeable plants were marked for reference in subsequent collections, and these plants were resampled at each subsequent visit. Symptoms, phenology, and plant age were recorded for each plant sampled. Plant age was rated according to the number of ramification orders, since lateral buds develop into new branches when plants resume vegetation after the unfavourable seasons of winter and summer. For analyses, plants were divided into four age classes. Class 1 included plants that had germinated recently and showed no ramification (<1 year), class 2 comprised those of 1 and 2 years, class 3 comprised those of 3, 4, and 5 years, and class 4, those of >5 years.

Samples consisted of four to six leaves from different branches if the plant had more than one, and of seeds for plants with ripe capsules. Each collection of leaves from an individual plant was placed in a plastic bag that was sealed and transported on ice to the laboratory, where leaf sap was extracted and the remaining material was stored at −80°C. Ripe capsules were also collected in plastic bags, and seeds were cleaned in the laboratory and stored in paper bags at 4°C. Leaf sap extracted from individual samples was used to inoculate leaves of *Nicotiana tabacum* L. cv- Xanthi-nc plants, which carry the *N* gene of hypersensitive resistance to most tobamoviruses that infect Solanaceae. Development of necrotic local lesions (NLL) in Xanthi-nc leaves indicated *Tobamovirus* infection.

Field surveys in pepper crops grown in plastic greenhouses in the province of Almería were done between 1984 and 2004, and are fully described in Fraile et al. (2011). Briefly, whenever *Tobamovirus* outbreaks occurred, symptomatic plants (3–5 per greenhouse) were randomly sampled in different, randomly chosen, greenhouses (8–10 per year). Leaf samples were handled as described for *N. glauca*. TMGMV outbreaks were frequent between 1992 and 2004, after which time the generalization of resistant pepper cultivars resulted in a reduction of the incidence of tobamoviruses (Fraile et al. 2011).

Data on the effective water deficit in the sampling area were calculated from daily values of Penman-Monteith evapotranspiration (Allen et al. 1998) and effective precipitation, available for Almería (36° 50′ 07″ N, 02° 24′ 08″W) at https://servicio.mapa.gob.es/websiar/SeleccionParametrosMap.aspx?dst=1. Daily data were aggregated by month.

### Characterization of TMGMV genotypes in field samples and estimation of seed transmission rates

Total RNA was extracted from leaves of field-sampled *N. glauca* plants with Trizol (NZYol, NZYtech, Lisbon, Portugal). For plants that had shown to be infected by tobamoviruses in the Xanthi-nc NLL assay, RNA extracts were analysed by reverse transcription (RT) and polymerase chain reaction (PCR) (RT-PCR) using first-strand cDNA synthesis kit (NZYtech, Lisbon, Portugal) and Phusion polymerase (Thermo Fisher Scientific, MA, USA), with specific primers that amplify the coat protein gene and the 3’UTR, TMGMVuRNA3, identical to nucleotide (nt) positions 5562–5590 of TMGMV M34077, and CGM3, complementary to the 3′ end (positions 6338–6355 of TMGMV M34077) (Bera et al. 2018). Agarose gel electrophoresis of RT-PCR amplicons confirmed if the infecting *Tobamovirus* was TMGMV and allowed the differentiation between Long-3’UTR and Short-3’UTR isolates (Supporting Fig. S1). Quantification of virus titre in field samples was performed by quantitative RT-PCR (RT-qPCR) as in (Fraile et al. 2014), using primers corresponding or complementary to positions 5969–5990 and 6037–6058 of the TMGMV genome (accession no. M34077). For each sample, 100 ng of total RNA were utilized with Brilliant III Ultra-Fast SYBR Green QRT-PCR Master Mix (Agilent Technologies, CA, USA) according to the manufacturer’s recommendations in a final volume of 10 μl. Assays were performed in duplicate on a LightCycler 480 II real-time PCR system (Roche, IN, USA). Levels of viral RNA were deduced from comparison with standard curves produced using a set of serial dilutions in water of purified virion RNA.

Transmission of TMGMV through the seed was quantified in seeds collected in the field from sampled plants. Five non-infected field mother plants, seven infected with TMGMV Long-3’UTR isolates, and seven infected with TMGMV Short-3’UTR isolates, were randomly chosen. Seeds were sown on sterile substrate, and 102 seedlings per plant were analysed in groups of three. Total RNA was extracted with Trizol and TMGMV was detected in RNA extracts using dot blot hybridization (Pagán et al. 2014) with a digoxigenin (DIG)-labelled 370 nt RNA probe complementary to positions 5730–6100 of a full-length cDNA clone derived from TMGMV isolate Ng 92/73 (MH730970.1). Hybridisation of the probe to samples was detected with anti-DIG antibodies (Fab, Roche, IN, USA) together with a chemiluminescent substrate for alkaline phosphatase (CSPD, Roche, IN, USA). Transmission probability was estimated for each of the 19 mother plants assuming a binomial distribution, as *P* = 1 − (*q*)^1/*k*^, where *P* is the probability of virus transmission by a single seed, *q* is the proportion of negative seedling pools analysed per plant, and *k* = 3 is the number of seedlings per pool (Gibbs and Gower 1960).

### Virus isolates, plant inoculation, and virus quantification

For experiments under controlled conditions, fourteen TMGMV isolates from *N. glauca* plants collected in SE Spain were used. These isolates were randomly sampled from our isolate collection with the condition that seven isolates had a Long-3’UTR (isolates Ng 96/5, Ng 94/6, Ng 92/73, Ng 96/11, Ng 99/20, Ng 99/11and Ng 99/15; in some experiments inoculation with isolate 96/11 failed and was substituted by Ng 96/25) and seven had a Short-3’UTR (isolates Ng 90/5, Ng 90/8, Ng 96/19, Ng 99/16, Ng 96/16, Ng 89/8, and Ng 89/15). Multiplication of these TMGMV isolates and purification of virus particles was simultaneous, and was as in (Bera et al. 2018). Assays were performed in plants of *N. glauca* grown from seeds collected in wild stands of this species in SE Spain and pooled. Plants were grown in 15 cm diameter, 1.5 L pots at 23–25°C and 16 h light photoperiod in a greenhouse, or at the same photoperiod and different constant temperatures in growth chambers, according to the assay. Plants were inoculated in the first two true leaves with 400 ng of purified virus particles suspended in 0.1 M phosphate buffer pH 7.2. Three 5 mm diameter samples from three systemically infected leaves were taken at different days post inoculation (dpi) according to the experiment, and total RNA was extracted. At the end of the experiment, plants were harvested, and the dry weight of the above-ground parts was determined. All experiments involved 10 replicated plants per treatment, and treatments were randomized. Inoculation success was 75% across isolates, and the number of infected plants per treatment varied between four and nine.

To test the effect of TMGMV infection on the performance of *N. glauca* under drought conditions, an experiment was carried out in a greenhouse in which the seven isolates of a Long-3’UTR and seven of a Short-3’UTR above were inoculated in *N. glauca* plants. Inoculation with isolate 96/11 of a Long-3’UTR failed. Plants were watered to field capacity until seven dpi. At this time, samples from systemically infected leaves were taken to quantify virus accumulation, and inoculated and mock-inoculated plants were divided into two sets: in one set, watering was stopped (water restriction group) and in the other watering continued (constant watering group). After seven dpi, plants were monitored daily and the onset of wilting of shoot apices was recorded. When 35% of all plants of the water restriction group showed wilted apices (17 days after watering withdrawal, DAWW), watering was resumed, and wilting symptoms, recovery, or mortality of the plants were monitored daily until 32 dpi. At this time, tissue samples were collected from plants of the constant watering group for TMGMV quantification, all plants were harvested, and plant dry weight was measured.

In all assays, viral multiplication was estimated from viral RNA accumulation in systemically infected leaves by RT-qPCR as described above. RT-PCR assays in RNA extracts of individual plants were performed to confirm the size of the 3’UTR at the end of the experiment.

### Genetic diversity and population genetic analyses

For genetic analyses, amplicons of 811 nt of the 126 kD protein gene and of 790–918 nt (depending on whether the isolate had a Short or a Long 3’UTR) of the CP gene plus the 3’UTR, were obtained using primers TobPimRepF, identical to nucleotides 439–463 of TMGMV M34077 and TobPimRepR, identical to nucleotides 1274–1296 of TMGMV M34077 (Fraile et al. 2011), and TMGMVuRNA3 and CGM3. Sanger determination of amplicon nucleotide sequences was outsourced to Stab Vida (Lisbon, Portugal). After manual curation of the sequences in MEGA X (10.0.5), concatenate sequences of 715 nt in the 126 kDa protein ORF and 425 nt in the coat protein ORF were aligned using MAAFT, version 7, and manually curated in MEGA-X (10.0.5) (Kumar et al. 2018). Genetic diversity of the viral population was estimated as *π*_T_ (Tajima 1983). The average within-population diversity, *π*_S_, was estimated for the variously defined subpopulations using the appropriate nucleotide substitution model and 1000 bootstrap replicates, and the interpopulation diversity was computed as *δ*_ST_ = *π*_T_—*π*_S_ (Nei and Kumar 2000). Population structure according to time, site, or host was estimated by the fixation index *N*_ST_ = δ_ST_/π_T_ (Nei 1973). Values of these parameters were calculated in MEGA-X (10.0.5). Effective population size*, N*_e_, was estimated as *N_e_ =* ***Θ/***2 *μ* where *Θ* = (*π*/1-*π*), genomes are considered as haploid, and *μ* is the mutation rate expressed as substitutions per site per year with a value of 3.8·10^−4^ (Fraile et al. 2011). The number of migrants among subpopulations was estimated as *XN_e_m* (Mills and Allendorf 1996), where *X* is 2 as we consider viruses as haploids, and *m* is the immigration rate per generation, considering generation as 1 year. To calculate *m*, a Metropolis-Hastings algorithm was used to explore all possible genealogies, as implemented in migrate-n (5.0.4) (Beerli et al. 2019).

### Statistical analyses

Data on the number of plants infected in the field by Long-3’UTR or Short-3’UTR isolates, number of symptomatic plants in the field, data to determine seed transmission rates and data on the number of wilted or surviving plants after a drought period were analysed by contingency tests. Data of viral accumulation in experiments, plant weight, and seed germination were all analysed similarly. Outliers were removed using Grubbs’ test and the distribution of the data was evaluated by means of Shapiro–Wilk test. If data did not follow a normal distribution, QQ-plots, histograms, and probability curves were adjusted to the data to find the best fit by Akaike’s Information Criterion (AIC) using the R package risk distributions. Homogeneity of variance was analysed with a Levene’s test. After this, full factorial generalized linear models (GLM) and post-hoc analyses for multiple comparisons (Fisher’s Least Significant Difference (LSD) or Analyses of Single Proportions, depending on the data distribution) were performed. Data of virus accumulation in field plant samples, or in experiments involving plant sampling at different times, were analysed using a generalized linear mixed model (GLMM), considering the collection date as a repeated measure and the 3’UTR size as a fixed factor, and the Wilcoxon signed-rank test was employed as a post-hoc analysis to identify specific pairwise differences between accumulation values. All statistical analyses were performed using R (4.3.1) (R Core Team, 2023) or IBM SPSS Statistics Version 29.0.0.0 (241).

## Results

### Dynamics of TMGMV infection in *N. glauca* populations

Seven collections were performed in six *N. glauca* populations (Fig. 1A, B) between February 2018 and 2020. The number of plants sampled per population at each date, and the number of plants infected by TMGMV isolates with a Long-3’UTR or a Short-3’UTR or by both types of isolates, as detected by RT-PCR (Supporting Table S1), are summarized in Table 1 and Fig. 1C. TMGMV prevalence (number of RT-PCR-positive plants over total sampled plants) was 0.56 (0.44–0.69), and it varied along the sampling period, as well as the relative frequency of plants infected by Long-3’UTR and Short-3’UTR isolates (Fig. 1C). Plants mixed-infected by Long-3’UTR and Short-3’UTR isolates were 3.52% of infected plants. Less than half of infected plants (39%) showed symptoms, which were of mild mosaic. The fraction of symptomatic plants varied according to the collection date (${\chi}_6^2$ = 54.988, *P* < .001), being highest in late winter (52% in 2018 and 2019, 71% in 2020 February collections), and lowest after the summer (~30%).

**Table 1:** Number of sampled plants, and of plants infected by Long-3’UTR, Short-3’UTR isolates or mixed infected by both types of isolates, per *Nicotiana glauca* population. Data are aggregated over collection times

**Population**	**Not infected**	**Infected by**				**Total**
		**TMGMV**	**Long-3’UTR**	**Short-3’UTR**	**Mixed infected**	
San José	112	31	31	0	0	143
La Datilera	23	117	117	0	0	140
Rambla Morales	10	52	49	1	2	62
Rambla Amoladeras	95	71	34	28	9	166
AL3115	53	37	25	10	2	90
El Edar	19	90	4	85	1	109
Total	312	398	260	124	14	710

**Figure 2:**
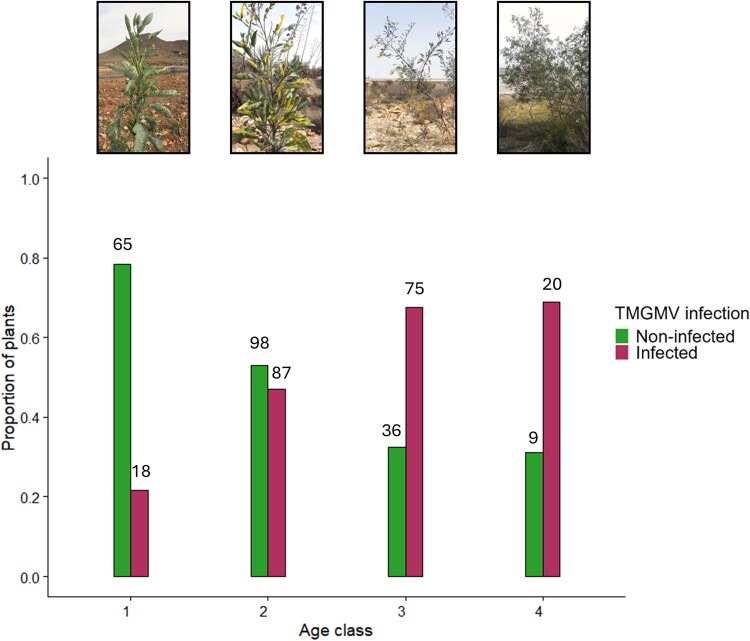
**Age structure and infection status of plants in the *N. glauca* population**. Data of the proportion of non-infected and infected plants were aggregated over collections, excluding plants resampled in successive collections, except if the resampled plants changed age class or infection status. Figures above columns indicated the number of non-infected or infected plants. *N. glauca* plants, representative of each age class, are shown at the top. Age classes are defined in materials and methods.

The frequency of TMGMV infection was analysed in relation to the demography of the host population. Plants that were resampled at different collection times were excluded from the analysis, except if they changed age class or infection status in successive collections. The Datilera population was excluded from this analysis as it was not possible to identify resampled plants. Figure 2 shows the proportion of infected and non-infected plants aggregated by collection and Supporting Table S2 shows the number of plants per population. The fraction of infected plants varied according to age class (${\chi}_3^2$ = 45.004, *P* < .001 in a contingency test), with prevalence increasing from 0.22 in age class 1 to 0.47 in age class 2 and 0.68–0.69 in age classes 3 and 4 (Fig. 2). The highest prevalence at age classes 3 and 4, together with most infections occurring at the transition between age classes 1 and 2, may suggest a higher survival of infected than non-infected plants, but to support this hypothesis, it is necessary to consider the number of plants that became infected by horizontal transmission. In other words, the analyses should be based on the number of plants at each age class that were infected by vertical transmission. The prevalence in age class 1 must be due, at least in part, to vertical transmission, and any new infection in the other age classes must be due to horizontal transmission. Rates of horizontal transmission at age classes 2 and higher can be estimated from the analysis of plants that were re-sampled during subsequent collections. Out of a total of 81 resampled plants, 51 were infected since the first collection, and 30 were not infected at the first collection, of which 12 were infected at later collections, which allows estimating a horizontal transmission rate of 0.40. Using this rate, we can estimate the number of horizontally infected plants in each age class, and subtracting it from the total number of infected plants at each age class to estimate the number of vertically infected plants, which are 52, 45 and 12, for age classes 2, 3, and 4, respectively, rather than the total number of 87, 75, and 20 shown in Supporting Table S2. With these new data, contingency tests showed that the fraction of infected plants varied according to age class (${\chi}_3^2$ = 23.970, *P* < .0001), increasing from age class 1 to age class 2 (${\chi}_1^2$ = 3.690, *P* = .0540) and from age classes 2 to 3 (${\chi}_1^2$ = 8.580, *P* = .0034) but not for age classes 3 to 4 (${\chi}_1^2$ = 0.0100, *P* = .9203). Similarly, we can estimate an upper threshold for the vertical transmission rate from the prevalence at age class 1, which is 0.22. Multiplying this rate by the number of plants in subsequent age classes, we can estimate the number of vertically infected plants for age classes 2, 3, and 4 at 46, 51, and 14, respectively. Again, contingency analyses showed that the fraction of infected plants varied according to age class (${\chi}_3^2$ = 32.32, *P* < .0001), not differing from age class 1 to age class 2 (*χ*^2^_1_ = 2.250, *P* = .1336) and increasing from age classes 2 to 3 (${\chi}_1^2$ = 14.770, *P* = .0001) but not for age classes 3 and 4 (${\chi}_1^2$ = 0.040, *P* = .8415). Thus, regardless of the approach used to estimate the effect of horizontal transmission, data are compatible with a higher survival of infected plants, which occurs at the transitions from age class 2 to subsequent age classes. Note that despite the estimates of both horizontal and vertical transmission being expected to have substantial errors, they yield consistent estimates of the number of vertically infected plants at each age class.

Figure 1 and Table 1 also show the relative frequency of plants infected by Long-3’UTR or Short-3’UTR isolates, and of plants infected by both types. The relative frequency of plants infected by Long-3’UTR isolates was about twice that of plants infected with Short-3’UTR isolates, although values varied over time. The fraction of plants infected by Long-3’UTR or Short-3’UTR isolates did not depend on the age class (${\chi}_3^2$ = 3.890, *P* = .274).

**Figure 3:**
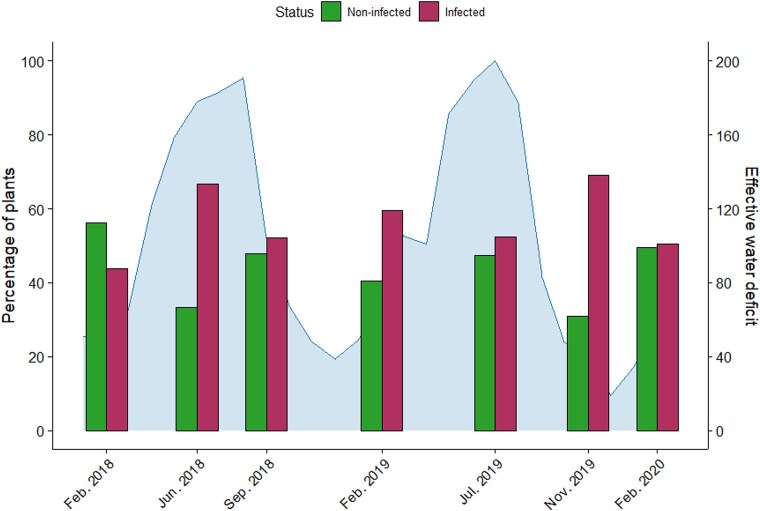
**Infection status of plants in the *N. glauca* population at the different collection times**. Infection data were aggregated over age classes, and all sampled plants were included. Proportions are relative to the total number of plants sampled at each collection time. Variation over the studied period of the monthly effective water deficit is shown shadowed.

The temporal change in the number of infected or non-infected plants was also analysed, including all sampled plants, as plants resampled over successive collections were computed as survivors. Figure 3 and Supporting Table S3 show the number of infected and non-infected plants aggregated over age classes. The fraction of infected plants varied according to collection (${\chi}_6^2$ = 19.910, *P* = .003), due to the comparisons between the collections of February 2018 and June 2018 (${\chi}_1^2$ = 8.938, *P* = .003), February 2019 and July 2019 (${\chi}_1^2$ = 5.519, *P* = .018), July 2019 and November 2019 (${\chi}_1^2$ = 6.682, *P* = .010), and November 2019 and February 2020 (${\chi}_1^2$ = .490, *P* = .006). As the age class structure of the collections of February 2018 and June 2018, and of July 2019 and November 2019 did not differ (${\chi}_3^2$ = 1.717, *P* = .633; ${\chi}_3^2$ = 1.441, *P* = .696, respectively), these data are consistent with a higher survival of infected than non-infected plants at specific periods of the year. We quantified the effective water deficit in the sampling area, as a possible factor causing plant stress in the semi-arid conditions of the study area (Fig. 3). There was a water deficit during the whole study period, particularly severe (higher than 100 mm) each year from May to August, that is, partially overlapping with the periods between the collections in which a decrease in non-infected plants and an increase in infected plants occurred (Fig. 3). This result may suggest that TMGMV-infected *N. glauca* plants are more tolerant to drought than non-infected plants.

### Differential accumulation of Long-3’UTR and Short-3’UTR TMGMV isolates in *N. glauca* plants

**Figure 4:**
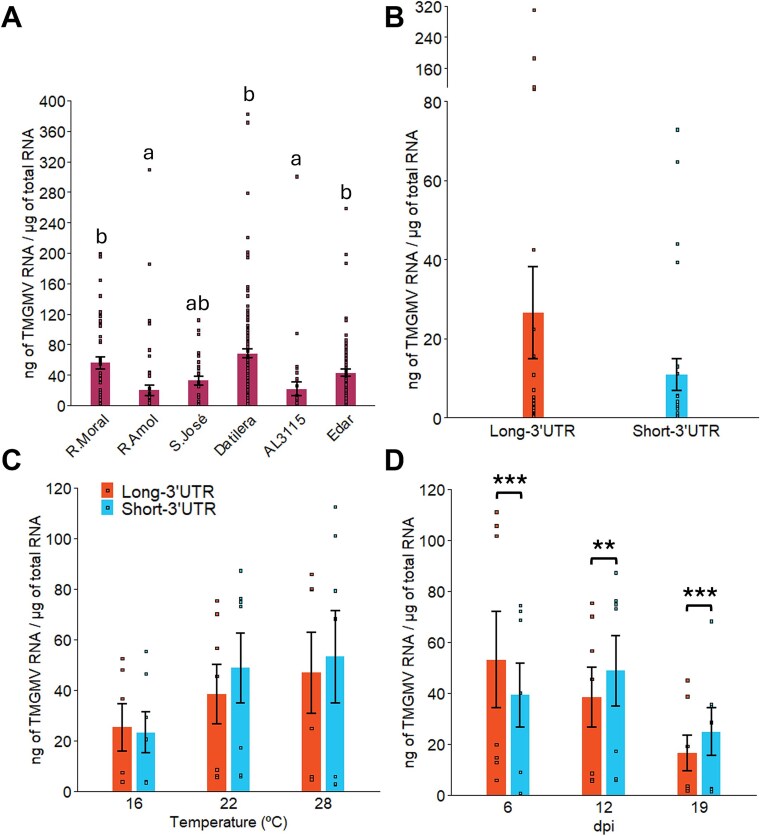
**Accumulation of TMGMV Long-3’UTR and Short-3’UTR isolates in *N. glauca* under field conditions (A, B) or under experimental conditions at different temperatures (C) and times after inoculation (D).** Data are shown as mean and standard error over the different numbers of infected plants per collection (Table S1, panels A and B) or as mean and standard error of six or seven replicated Long-3’UTR or Short-3’UTR isolates (panels C and D). Data in panel A are aggregated for Long-3’UTR and Short-3’UTR isolates and letters over columns indicate the significance of differences in virus accumulation; data in panel B are from the Rambla Amoladeras population, aggregated over collection times; data in panel C are for different temperatures and 12 dpi, and data in panel D are for plants grown at 22°C constant temperature at different dpi. Asterisks indicate differences at the 0.01 (**) or 0.001 (***) level. R. moral, Rambla Morales. R. amol, Rambla Amoladeras.

The lower frequency of plants infected by Short-3’UTR than by Long-3’UTR isolates in the *N. glauca* population (Fig. 1) could be due to a lower multiplication of Short-3’UTR isolates, as contact transmission of tobamoviruses depends on virus titre (Sacristán et al. 2011). Thus, the accumulation of TMGMV was quantified by RT-qPCR in RNA extracts from leaves of the sampled plants that were RT-PCR positive for either type of TMGMV isolate. However, the distribution of Short-3’UTR isolates was limited to some populations and collection dates (Supporting Tables S1 and S4), it was necessary to analyse if virus accumulation varied among the *N. glauca* populations. Accumulation data followed a gamma distribution, and a GLMM considering population as a fixed factor, collection date as a repeated measure and the population per sampling date interaction, showed a significant effect on virus accumulation of the population (*F*_5,345_ = 7.669, *P* < .001), the sampling date (*F*_6,344_ = 8.963, *P* < .001) and their interaction (*F*_29,321_ = 3.457, *P* < .001) (Fig. 4A). Thus, comparison of the accumulation of Long-3’UTR and Short-3’UTR isolates was restricted to Rambla Amoladeras, the only population where both types of isolates were consistently found. A paired Wilcoxon test showed that the accumulation of Long-3’UTR and Short-3’UTR did not differ when data were aggregated across collection date (*V* = 125, *P* = .325) (Fig. 4B) or analysed separately at each collection date (*W* < 14 000, *P* > .143 in non-paired Wilcoxon’s tests).

The differences in accumulation of TMGMV according to population and collection time suggest an effect of environmental conditions on TMGMV accumulation. To test this hypothesis, the accumulation of seven isolates of a Long-3’UTR and seven of a Short-3’UTR in *N. glauca* plants was analysed in growth chambers at constant temperatures of 22°C, i.e*.* the mean temperature at the field sites during the 2-year period, and at 28°C and 16°C, close to the mean maximal and mean minimal temperatures of the period. Virus accumulation in systemically infected leaves was quantified at twelve dpi for assays at 16 and 28°C, and at 6, 12, and 19 dpi for the assay at 22°C. In the 16 and 28°C assays, inoculation with isolate 96/11, of a Long-3’UTR, failed, and this isolate was substituted for 96/25 in the assay at 22°C. Results are shown in Fig. 4 C and D and Supporting Table S5. Viral accumulation at 12 dpi at the three temperatures was analysed by a GLM considering temperature and 3’UTR size as fixed factors, virus isolate nested to 3’UTR size, and the temperature interaction per 3’UTR size. There was an effect of temperature (*F*_2,297_ = 23.623, *P* < .001), 3’UTR size (*F*_1,297_ = 4.353, *P* = .038) and isolate nested to 3’UTR size (*F*_12,297_ = 89.140, *P* < .001) on virus accumulation, but not of the 3’UTR size per temperature interaction (*F*_2,297_ = 1.442, *P* = .238).

The kinetics of the accumulation at 22°C of Long-3’UTR and Short-3’UTR isolates was studied for a 19-dpi period. A GLMM considering 3’UTR size as a fixed factor, isolate nested to 3’UTR size, the sampling time as a repeated measure and the 3’UTR size per sampling date interaction showed that the 3’UTR size did not have an effect on virus accumulation (*F*_1,335_ = 0.110, *P* = .740), while isolate nested to 3’UTR size (*F*_12,324_ = 27.410, *P* < .001), sampling time (*F*_2,334_ = 30.561, *P* < .001) and the interaction between 3’UTR size and sampling time (*F*_2,334_ = 9.609, *P* < .001) did. GLMs considering 3’UTR size as a fixed factor and isolate nested to 3’UTR size for each time after inoculation showed that at each time there was a significant effect of 3’UTR size (*F*_1,88_ = 13.831, *P* < .001; *F*_1,91_ = 7.918, *P* = .006; *F*_1,94_ = 14.273, *P* < .001, for 6, 12, and 19 dpi, respectively) and of isolate nested to 3’UTR size (*F*_12,88_ = 35.165, *P* < .001; *F*_12,91_ = 38.652, *P* < .001; *F*_12,94_ = 17.050, *P* < .001, for 6, 12, and 19 dpi, respectively). Thus, Long-3’UTR isolates accumulated to higher levels early post-infection, and to lower levels later, than Short-3’UTR isolates.

In conclusion, the relative accumulation of TMGMV isolates with a Long-3’UTR or a Short-3’UTR in *N. glauca* plants depends on the temperature and the time after infection.

### Effect of TMGMV infection on the performance of *N. glauca* plants under drought conditions

We analysed if TMGMV infection improved drought tolerance in *N. glauca*, which could be relevant in the semi-arid environment of *N. glauca* populations in SE Spain, and if this effect was different for Long-3’UTR and Short-3’UTR isolates. For this inoculated and mock-inoculated plants were divided into two sets: a water restriction group, and a constant watering group.

Virus accumulation at 7 and 32 dpi in plants under constant watering (Supporting Fig. S2 and Supporting Table S6) was consistent with assays at constant temperatures: a GLMM considering 3’UTR size as a fixed factor, isolate nested to 3’UTR size, the sampling time as a repeated measure and the 3’UTR size per sampling date interaction, showed significant effects on virus accumulation of 3’UTR size (*F*_1,305_ = 81.264, *P* < .001), time after inoculation (*F*_1,305_ = 24.653, *P* < .001), the interaction of both factors (*F*_1,305_ = 127.924, *P* < .001), and of isolate nested to 3’UTR size (*F*_11,294_ = 45.462, *P* < .001). Long-3’UTR isolates accumulated to higher levels at 7 dpi, but not at 32 dpi, than Short-3’UTR isolates (*F*_1,176_ = 290.344, *P* < .001; *F*_1,95_ = 2.982, *P* = .087 in GLMs considering 3’UTR size as a fixed factor and isolate nested to 3’UTR size for 7 and 32 dpi, respectively).

Plant biomass was quantified as dry weight at 32 dpi, and the effect of infection on plant growth relative to mock-inoculated controls was analysed (Supporting Fig. S3 and Supporting Table S7). Separate GLMs were conducted for the water restriction and constant watering treatments, with treatment (plants mock-inoculated, infected by Long-3’UTR isolates or infected by Short-3’UTR isolates) as a fixed factor and isolate nested to treatment. For plants under constant watering, plant dry weight significantly depended on treatment (*F*_2,104_ = 8.264, *P* < .001) but not on isolate nested to treatment (*F*_11,104_ = 1.246, *P* = .264), dry weight was higher for mock-inoculated plants than for plants infected by either Long-3’UTR or Short-3’UTR isolates (*P* < .000), for which weight did not differ (*P* = .865) in a LSD test. For the water restriction group, plant dry weight depended on both treatment and isolate nested to treatment (*F*_2,81_ = 5.508, *P* = .005; *F*_11,81_ = 2.533, *P* = .008, respectively). Again, dry weight was higher for mock-inoculated plants than for plants infected by either Long-3’UTR or Short-3’UTR isolates (*P* = .002 in both comparisons), for which weight did not differ (*P* = .970). Thus, infection by both types of TMGMV isolates had a negative effect on the growth of *N. glauca* plants, whether the plants were under conditions of water stress or not.

**Figure 5:**
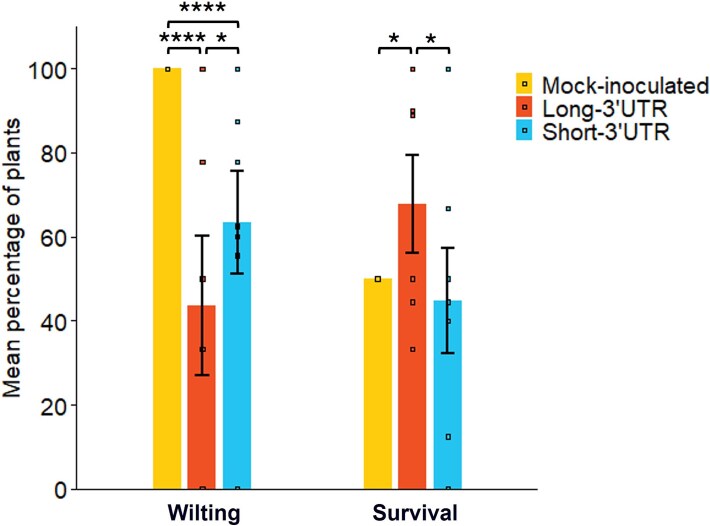
**Effect of TMGMV infection on wilting and survival of *N. glauca* plants under drought conditions.** Proportion of plants mock-inoculated or infected with TMGMV Long-3’UTR or Short-3’UTR isolates showing wilted apices at 20 DAWW, or surviving at 25 DAWW, that is, at 8 days after watering was resumed. Data for infected plants are mean and standard error of six (Long-3’UTR) or seven (Short-3’UTR) replicated isolates. Asterisks indicate differences at the 0.05 (*) or 0.0001 (****) level.

The effect of drought on mock-inoculated or infected plants was assessed by monitoring the onset of wilted shoot apices and the number of plants surviving at the end of the experiment. Shoot apex wilting appeared only under water restriction, earlier in the mock-inoculated than in the TMGMV-infected plants, and earlier in plants infected by Short-3’UTR isolates than in plants infected by Long-3’UTR isolates. (Supporting Fig. S4, Supporting Table S8). The fraction of plants with wilted apices (Fig. 5) increased until 20 DAWW, 3 days after watering was resumed, and at this time it was 100% for mock-inoculated plants. The fraction of plants infected by Long-3’UTR or Short-3’UTR isolates that had wilted apices was compared with the null hypothesis of no difference with mock-inoculated plants by contingency tests, which showed that the fraction of plants infected by either type of isolate with wilted apices was smaller than for mock-inoculated plants (${\chi}_1^2$ > 23.750, *P* < .0001). Also, the proportion of plants infected by Long-3’UTR isolates with wilted apices was lower than that of plants infected by Short-3’UTR isolates (${\chi}_1^2$ = 5.580, *P* = .0182). At the end of the experiment, 50% of mock-inoculated plants had survived (Fig. 5, Supporting Table S10). Again, the fraction of surviving plants infected by either Long-3’UTR or Short-3’UTR isolates was compared with the null hypothesis of no difference with mock-inoculated plants by contingency tests, which showed that the fraction of surviving plants infected by Long-3’UTR was higher than that of mock-inoculated plants (${\chi}_1^2$ = 4.150, *P* = .0416) and that of plants infected by Short-3’UTR (${\chi}_1^2$ = 5.060, *P* = .0245), while the fraction of survivors did not differ between plants infected by Short-3’UTR isolates and mock-inoculated plants (${\chi}_1^2$ = 0.000, *P* = 1.000).

Thus, TMGMV infection by Long-3’UTR and Short-3’UTR delays wilting, and infection by Long-3’UTR, but not Short-3’UTR, increases survival of *N. glauca* plants under drought conditions.

### Seed transmission of TMGMV in *N. glauca*

**Figure 6:**
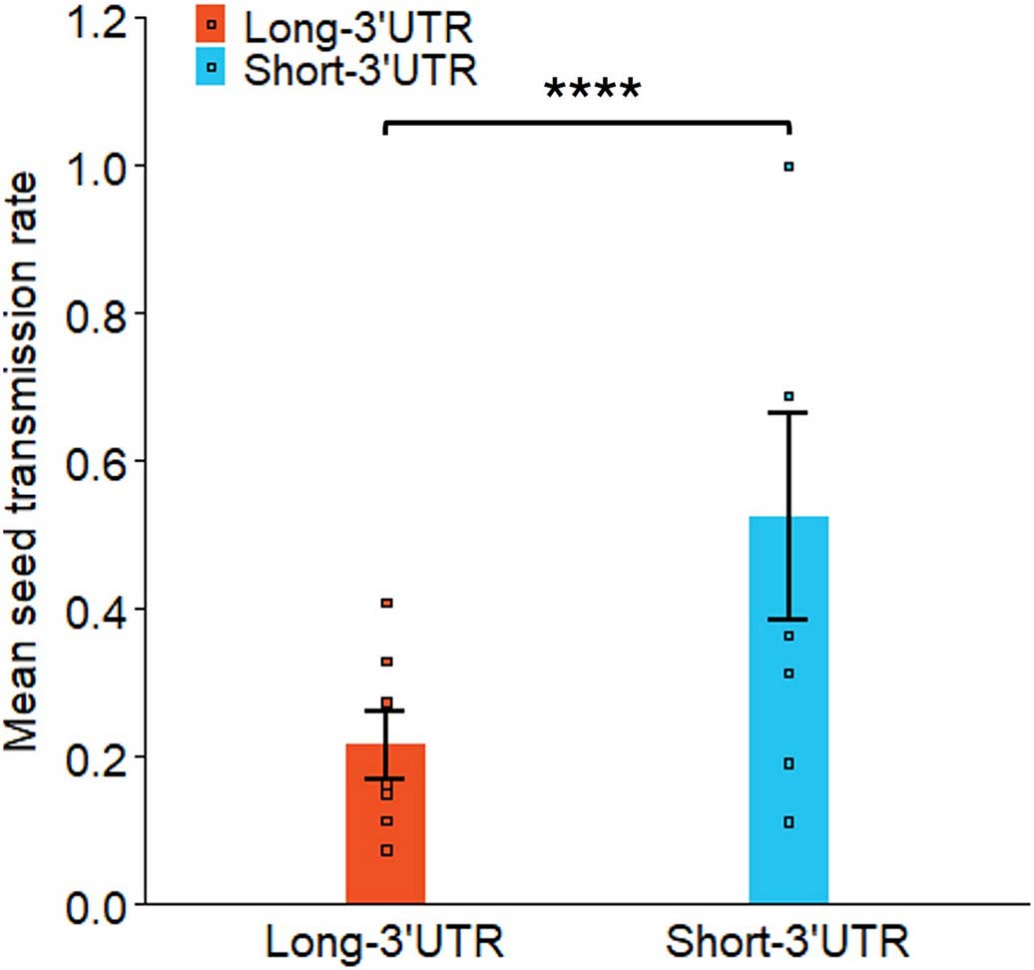
**Rates of transmission of TMGMV in *N. glauca* seeds.** Data are mean and standard error of seven field plants infected by either Long-3’UTR or Short-3’UTR isolates. Asterisks indicate differences at the 0.0001 (****) level.

Seed transmission of TMGMV was quantified in field-collected seeds from plants infected by Long-3’UTR or Short-3’UTR isolates, or not infected with TMGMV. Infection of the mother plants by either Long-3’UTR or Short-3’UTR isolates did not affect seed viability (*F*_2,14_ = 0.493, *P* = .620), which was always high (Supporting Table S11). Seed transmission probability was of 0.37 ± 0.083 when the length of the 3’UTR was not considered, and was lower for Long-3’UTR isolates (0.22 ± 0.05) than for Short-3’UTR (0.52 ± 0.14) (${\chi}_1^2$ = 29.830, *P* < .0001) (Fig. 6 and Supporting Table S12). TMGMV was not detected in any three-seedling pools of the 102 seedlings analysed from five non-infected plants.

### TMGMV population genetic structure in *N. glauca* and pepper crops

The genetic structure of the TMGMV population was analysed to understand the inoculum fluxes between the *N. glauca* wild reservoir and the pepper crop. Analyses were based on the concatenated nucleotide sequences of a 715-nt fragment in the 126 kDa protein ORF and a 425-nt fragment of the coat protein ORF. The *N. glauca* TMGMV population was represented by sequences from 48 TMGMV-infected plants from the first and last collections (February 2018 and 2020). These plants were randomly sampled, with no consideration for the 3’UTR size of the infecting TMGMV isolate. Their distribution across the six *N. glauca* populations is shown in Supporting Table S13. The genetic diversity of the TMGMV population was *π*_T_ = 0.0150 (0.0115%–0.0185 95% confidence interval, CI), and when two subpopulations were defined according to collection time, the fixation index was *N*_ST_ = 0.0004 (−0.0182%–0.0190 95% CI), indicating that the 48 sequences were not structured according to time of collection and could be treated as a single population.

When the six subpopulations were defined according to the six *N. glauca* populations, the fixation index *N*_ST_ = 0.2118 (0.1046%–0.3190 95% CI) indicated spatial structure. The *N*_ST_ values for each pair-wise comparison of subpopulations (Supporting Table S14A) showed a significant but low correlation (*r* = 0.274, *P* = .032) with the distance in km between those populations in a Mantel test (Supporting Table S14B), consistent with a hypothesis of isolation by distance. The effective population size, *N*_e_ was estimated for the six TMGMV subpopulations (Supporting Table S15) and was used to compute the number of migrants per generation (year) among the subpopulations (Supporting Table S16). The number of migrants per year (2.04–5.99) was consistent with the spatial structure indicated by the *N*_ST_ values.

Similarly, population structure, effective population sizes, and migration rates were estimated between the *N. glauca* and pepper TMGMV populations. For this, the sequences of fourteen TMGMV isolates from pepper crops collected between 1992 and 2004 and of twelve TMGMV isolates collected between 1989 and 1999 were used. The genetic diversity of these pepper and *N. glauca* TMGMV populations was similar (***π***_T_ = 0.0269, 0.0208%–0.0330 95% CI for pepper, ***π***_T_ = 0.0209, 0.0156–0.0262 95% CI for *N. glauca*), and the fixation index *N*_ST_ = 0.0434 (0.0232–0.0636 95% CI) showed no evidence of genetic structure associated with host plant species. The *N*_e_ value of both populations was similar (34.98 for pepper, 28.09 for *N. glauca*). The number of migrants per year was higher from the *N. glauca* to the pepper subpopulation (13.057) than contrary wise (3.345). When the 14 sequences from pepper isolates were compared with 18 sequences from *N. glauca* from 2018 to 2020, similar results were obtained (not shown).

## Discussion

Identifying traits that make a host a good reservoir is central to understanding infection dynamics, predicting emergences, and mitigating virus epidemics. In this study we analyse the traits that determine the potential of *N. glauca* as a reservoir of TMGMV for its emergence in pepper crops. *N. glauca*, phylogenetically related to pepper in the Solanaceae family, is an invasive species in many warm and dry regions of the world (Vélez-Gavilán 2023). *N. glauca* grows quickly in its early stages, with absolute growth rates up to 14 g dry mass/week, net assimilation rates of 29 g/m^2^/week, and leaf mass per unit area of 0.008–0.010 g/cm^2^ (Curt and Fernández 1990). Thus, when young, plants with these traits have a quick-return physiology (Wright et al. 2004, Cronin et al. 2010). Previous reports indicate *N. glauca* as a maintenance host of TMGMV: TMGMV was detected in *N. glauca* since the late nineteen century, and it had a high prevalence in all the regions where it has been analysed, and for extended periods of time (McKinney 1929, Randles et al. 1981, Rodríguez-Cerezo et al. 1991, Fraile et al. 1997, Bodaghi et al. 2004). TMGMV emerged in the early 1980s in pepper crops in SE Spain (Fraile et al. 2011), a major region of intensive pepper production in plastic greenhouses (Velasco et al. 2020) that share the space with *N. glauca* populations. Thus, a reasonable hypothesis is that spillover from *N. glauca* to pepper is at the origin of such emergence. Here we analyse the factors that determine the maintenance of TMGMV in *N. glauca* and the epidemiological connectivity between *N. glauca* and pepper populations to better understand what makes a plant a good reservoir for virus emergence in crops.

Consistent with earlier surveys (Rodríguez-Cerezo et al. 1991), our recent data showed a high prevalence of TMGMV in *N. glauca*. Infection did not occur randomly along the plant life span, but was highest in its early stages when plant growth is fast, consistent with predictions and analyses showing that fast growth is traded against resistance, resulting in higher transmission (Cronin et al. 2010, 2014, Johnson et al. 2012). The variation of the fraction of infected plants according to age class and time of collection is consistent with a higher survival of infected plants, and suggests higher survival might be associated, at least in part as other sources of stress were not analysed, with improved survival under drought conditions, which we demonstrate under greenhouse conditions. Improved drought tolerance of virus-infected plants is not universal across pathosystems (e.g. Olson et al. 1990, McLaughlin and Windham 1996), but it has been shown to occur in a variety of plant-virus interactions (Xu et al. 2008, Davis et al. 2015, Aguilar et al. 2017, Moreno et al. 2022). These results show that under experimental conditions, plant viruses may behave as conditional mutualists (Roossinck 2011, 2015, Lefeuvre et al. 2019, González et al. 2021) increasing tolerance to other stresses, which may be particularly relevant in the often-suboptimal conditions of wild plant communities in nature (Roossinck 2015, Fraile and García-Arenal 2016). Our data show that TMGMV infection has a negative effect on the growth of *N. glauca* plants under both constant water availability and drought, which could translate into reduced reproduction. However, under drought conditions and perhaps under other stresses as well, TMGMV infection with isolates of a Long-3’UTR will increase plant survival, thus having a beneficial effect on this component of the host fitness. The benefits to *N. glauca* of TMGMV infection depend on physical conditions of the environment is consistent with the definition of conditional mutualism (Cushman and Whitam 1989). The huge number [up to 10 000 and 1 000 000 (Vélez-Gavilán 2023)] of highly viable (Supporting Table S11) seeds produced per plant compared to the number of recruits (Fig. 2) suggests that TMGMV infection would have a small effect on the plant’s population dynamics, regardless of the environment-dependent balance of the effects on growth and survival. Thus, low or even negative virulence (i.e. conditional mutualism) of TMGMV is associated with, and may determine, its high prevalence in *N. glauca* populations (Hypothesis 1) and its role as a maintenance host.

A 147 nt duplication in the genome’s 3’UTR is involved in determining the differential within-host fitness of TMGMV isolates across hosts. Isolates without the duplication (Short-3’UTR) multiply to higher levels in pepper than Long-3’UTR isolates, while both types of isolates multiply to similar levels in *N. glauca* (Bera et al. 2018). Our present results show that in *N. glauca* populations Long-3’UTR isolates were twice as prevalent as Short-3’UTR isolates. Contact transmission of tobamoviruses depends on virus titre (Sacristán et al. 2011), but the lower frequency of Short-3’UTR isolates in the field is not explained by their lower accumulation, as Long-3’UTR isolates and Short-3’UTR isolates accumulated to similar levels in field plants. However, experiments under controlled conditions showed that the relative accumulation of Long-3’UTR and Short-3’UTR isolates depends on the temperature, and changes as infection in the infected plant progresses, a result that underscores the risk of using data from assays at constant conditions and considering a single time after inoculation to draw conclusions on across-host fitness trade-offs (García-Arenal and Fraile 2013, Elena et al. 2014) and to explain infection dynamics of multi-host viruses. Long-3’UTR isolates accumulate to higher levels early after infection than Short-3’UTR isolates, which would result in Long-3’UTR isolates outcompeting Short-3’UTR isolates in mixed infection, as reported (Bodaghi et al. 2004) and contribute to explain both their higher frequency in the field and the low frequency of plants mixed-infected by both types of isolates. It should be noted that a phylogeny based on the concatenated sequences of fragments of the 126 K and coat protein ORFs showed distinct clustering patterns. Isolates with a Short-3’UTR formed two distinct clusters that differed from the two separate clusters formed by isolates with a Long-3’UTR (Fig. S5). No recombinant between Long-3’UTR and Short-3’UTR was detected. Thus, neither Long-3’UTR or Short-3’UTR isolates are monophyletic. However, all isolates except Rambla Morales 40 share a common ancestor. These results are compatible with epistatic interactions between the 3’UTR and some unidentified region(s) in the rest of the genome, as was concluded by Bera et al. (2018). Our results also show that, in addition to being a host-specific determinant of virus multiplication, the 3’UTR duplication has pleiotropic effects on other components of the virus fitness (Hypothesis 2). Thus, Long-3’UTR isolates, but not Short-3’UTR isolates, have an effect of increasing the survival of infected plants under drought conditions, which would further contribute to their higher prevalence. On the other hand, Short-3’UTR isolates were seed-transmitted in *N. glauca* twice as efficiently as Long-3’UTR isolates, which would in part compensate for the advantage of Long-3’UTR isolates in competition and in plant survival in drought, and explain the maintenance of Short-3’UTR isolates in the *N. glauca* population. Contrasting (positive or antagonistic) pleiotropic effects of host-range mutations on different fitness components have been demonstrated for other tobamoviruses (Fraile et al. 2011, 2014, Bera et al. 2017), and are difficult to integrate into net fitness effects that would explain the dynamics of different genotypes in virus populations (Cuevas et al. 2024).

Population genetic analyses show a low genetic diversity for the TMGMV population in *N. glauca*, with values similar to previous reports (Rodríguez-Cerezo et al. 1991, Fraile et al. 1996), a low effective population size also in the order of earlier estimates (Moya et al. 1993), and weak evidence of spatial structure and isolation by distance. Similarly, the TMGMV population in SE Spain shows a low differentiation according to host, *N. glauca* and pepper, and high migration between host species. Also, migration from *N. glauca* to pepper will reduce the effects of purifying selection in this host. We cannot at present explain why migration was higher from *N. glauca* to pepper than *vice versa*, but it may be speculated that the temporal discontinuity of the pepper population may be a factor. Higher migration from *N. glauca* to pepper may explain why TMGMV pepper isolates are not adapted to pepper, while *N. glauca* isolates are adapted to *N. glauca* (Bera et al. 2018): the strong influx of *N. glauca* isolates, where Long-3’UTR prevails, prevents the fixation of the beneficial Short-3’UTR allele in the pepper population (Hypothesis 3). We did not survey other potential hosts in the area, except for the wild Solanacea *Solanum nigrum* L., in which we did not detect TMGMV infection (not shown). Still, we cannot discard that other TMGMV hosts might also have a role in shaping the genetic structure of TMGMV in pepper and *N. glauca*. Migration rates also show a high epidemiological connectivity between the pepper and *N. glauca* populations, a condition for the latter to act as a reservoir for emergence.

Thus, the results of this study significantly contribute to understanding what factors contribute to the reservoir potential of a host, showing that low virulence or conditional mutualism may contribute to the long-term maintenance of the virus in the reservoir population, and that pleiotropic effects of host range mutations may explain the maintenance in the reservoir population of genotypes that are fitter in the target host. Moreover, this study underscores that even in a simple system of two hosts and one virus, the effects of infection on a range of host life-history traits, and the effects of host range mutations on different components of virus fitness, need to be considered to understand the dynamics of infection, host range evolution, and virus emergence.

## Supplementary Material

de_Andres_et_al_Supplementary_Figures_Rev2_veaf044

de_Andres_et_al_Supplementary_tables_veaf044

## Data Availability

All data generated and analysed in this study are included in this article and its supporting information files. Newly determined nucleotide sequences and other nucleotide sequences analysed are available in GenBank under accession numbers PP622813-PP622929, MH730945–6, MH730950, MH730956–7, MH730961, MH730964–5, MH730967–8, MH730970; and in EMBL data bank accession numbers FN594805–10, FN594812–5, FN594817, FN594854–5, FN594857–8, FN594862–3, FN594865–6, FN594874.
