# Supplementary material for: What makes a host a good reservoir? Determinants of the reservoir potential of Nicotiana glauca for tobacco mild green mosaic virus
Source: Virus Evol. 2025 Jun 6;11(1):veaf044. doi: 10.1093/ve/veaf044 (PMC12203087; doi:10.1093/ve/veaf044)
Supplement: de_Andres_et_al_Supplementary_Figures_Rev2_veaf044 [file de_andres_et_al_supplementary_figures_rev2_veaf044.docx]

**SUPPORTING FIGURES**


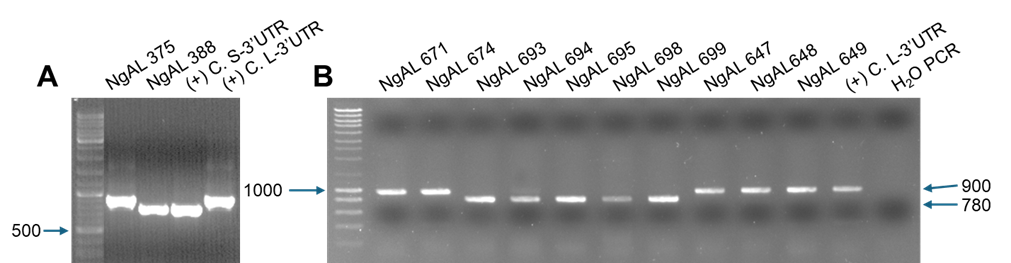


**Figure S1. Detection of 3’UTR dimorphism in agarose gel electrophoresis**. RT-PCR amplicons detecting Short- or Long-3’UTR dimorphism are shown. Panel A shows the different mobility of amplicons from controls for Long-3’UTR (C.L-3’UTR) and Short-3’UTR (C.S-3’UTR), which are isolates P98/12 and P94/29, respectively, compared to two field isolates from *N. glauca*. Panel B shows detections for different TMGMV isolates from *N. glauca*.


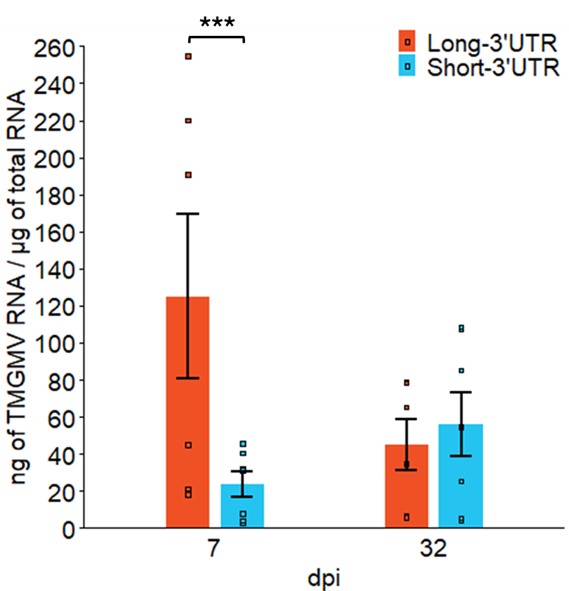


**Figure S2: Accumulation of TMGMV Long-3’UTR and Short-3’UTR isolates in *N. glauca* under experimental greenhouse conditions at different times after inoculation.** Data are for plants under full watering**.** Viral accumulation (ng of viral RNA / μg of total plant RNA) is shown as mean and standard error of six (Long-3’UTR) and seven (Short-3’UTR) replicated isolates.  Asterisks (***) indicate differences at the 0.001 probability level.


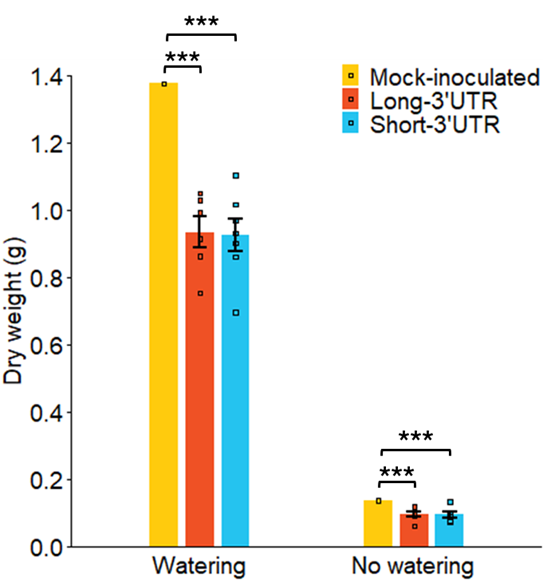


**Figure S3. Effect of TMGMV infection in *N. glauca* biomass at different water availabilities.** Data are presented separately for isolates with a Long-3’UTR and a Short-3’UTR genotype as mean dry weight (g) and standard error of six (Long-3’UTR) and seven (Short-3’UTR) replicated isolates.  Asterisks (***) indicate differences at the 0.001probability) level.


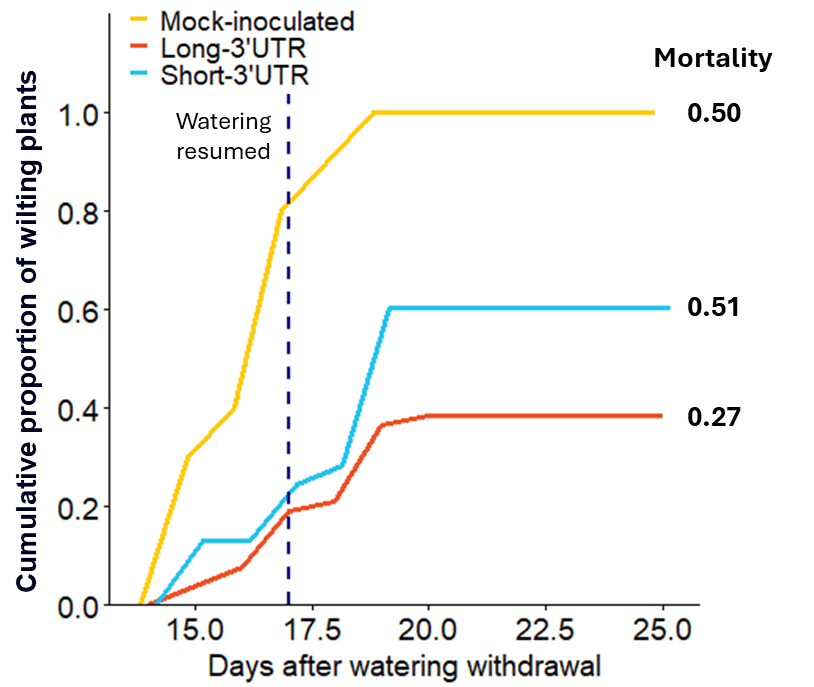


**Figure S4. Effect of TMGMV infection in *N. glauca* on the apparition of wilting symptoms under drought conditions.** Data are presented as percentage of plants that showed wilted apices at different days after watering withdrawal (DAWW).  The blue dash line represents the day in which watering was resumed. Figures by each line represent the mortality rate of each group at the end of the experiment.


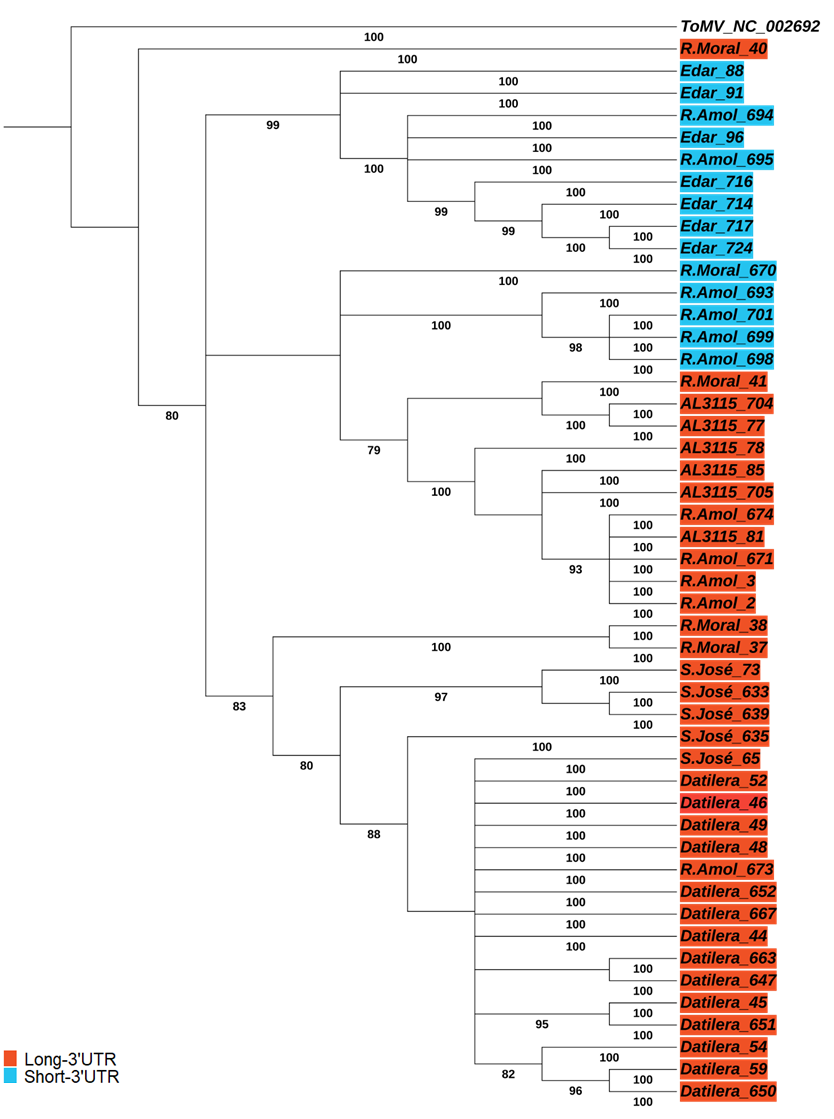


**Figure S5. Bayesian inference phylogeny of TMGMV isolates**. The phylogeny is based on the concatenated sequences of the 126 kDa protein and coat protein genes used in the population genetic analyses for 48 field isolates identified by the population name and a numeral, and by the size of the 3’UTR. Bayesian posterior probabilities higher than 70% are indicated. Datilera = La Datilera, Edar = El Edar ,R. Amol. = Rambla Amoladeras, R. Moral = Rambla Morales, S. José = San José.
